# Supplementary material for: Characterization of Expression and Epigenetic Features of Core Genes in Common Wheat
Source: Genes (Basel). 2022 Jun 21;13(7):1112. doi: 10.3390/genes13071112 (PMC9317296; doi:10.3390/genes13071112)
Supplement: Supplementary file 1 [file genes-13-01112-s001.zip › genes-1758854-supplementary/Supplementary Figures-proof.pdf]

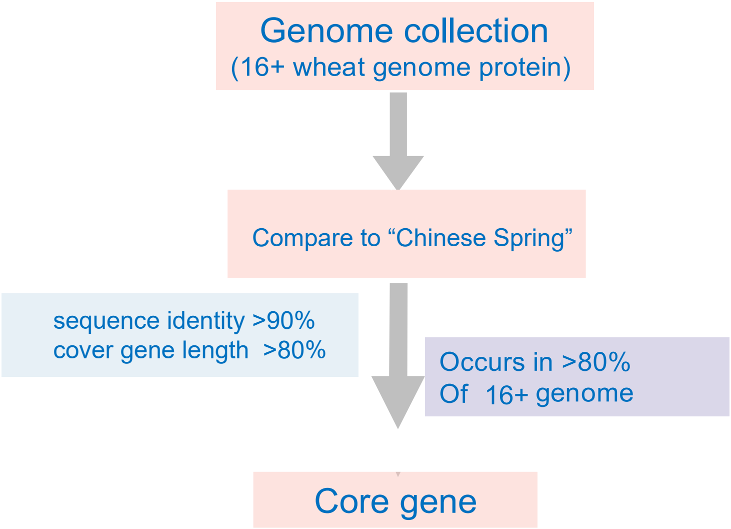

Figure S1. Flow chart for the identification of core genes in wheat

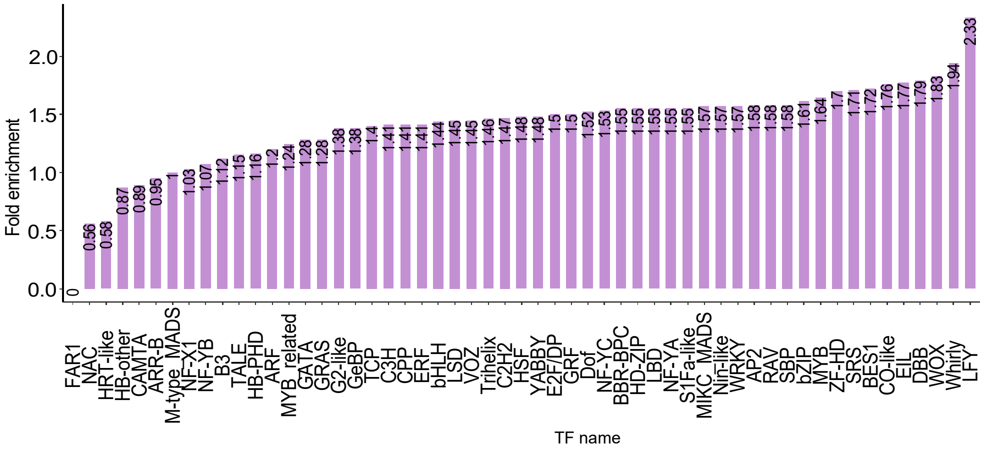

Figure S2. Transcription factor (TF) enrichment analyses of core genes, *x*-axis indicates the type of TFs, and *y*-axis indicates the fold change of TFs in the core genome compared to the corresponding the TFs in the whole genome.

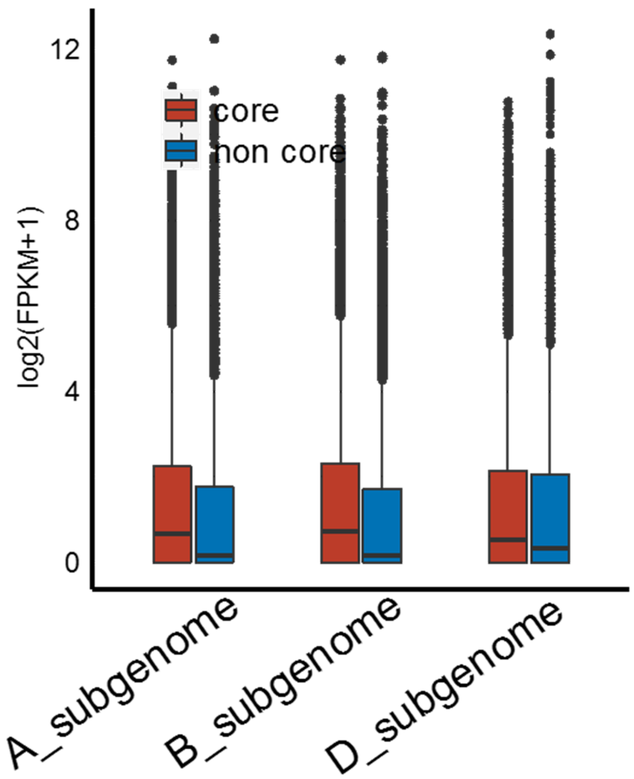

Figure S3. Comparison of expression levels of all core genes as well as non-core genes within and between subgenomes

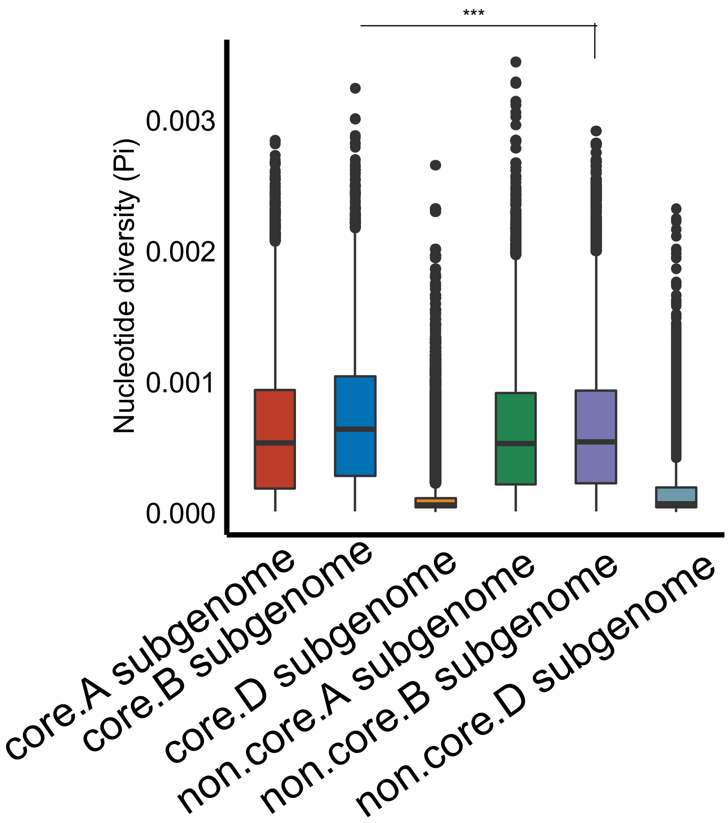

Figure S4. Comparison nucleotide diversity of all core genes as well as non-core genes within and between subgenomes. Where *** indicates that $p < 0.001$

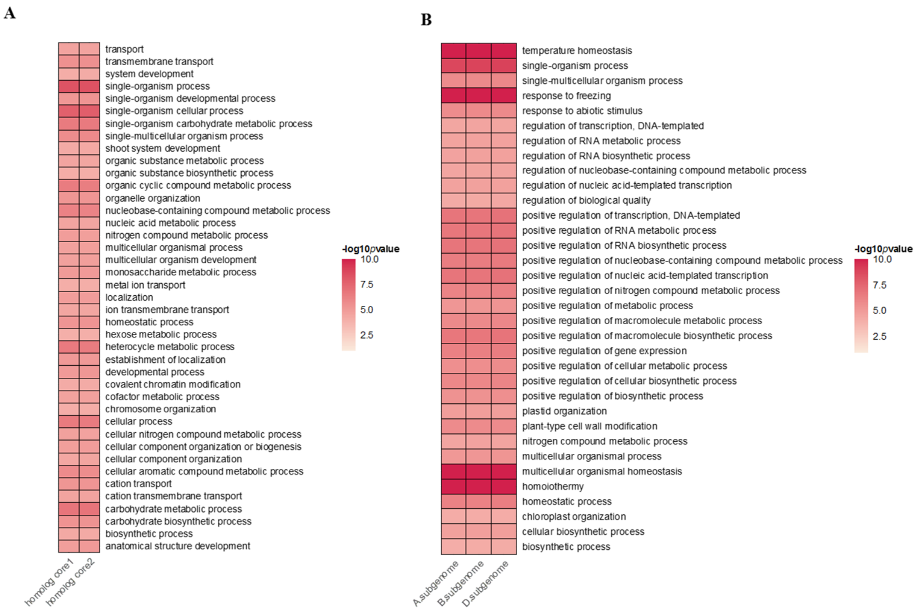

Figure S5. GO enrichment analysis of homologous gene pairs(A) and 1:1:1 genes(B) that are both core genes. These results demonstrate that homologous gene pairs are all core genes between them and that they are functionally conserved

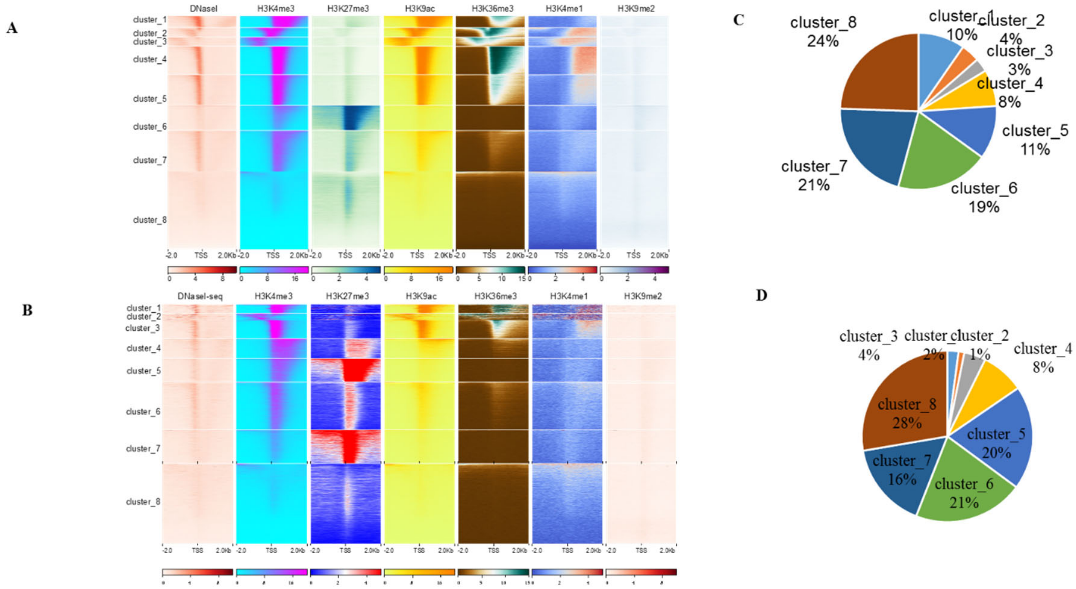

Figure S6. Genome-wide analyses of chromatin features of all genes.

Epigenetics landscapes of all genes (**A**) and non-expressed core genes (**B**). (**C**) The number and proportion of transcription factors corresponding to each chromatin feature. (**D**) The proportion of non-expressed core genes corresponding to each chromatin feature.

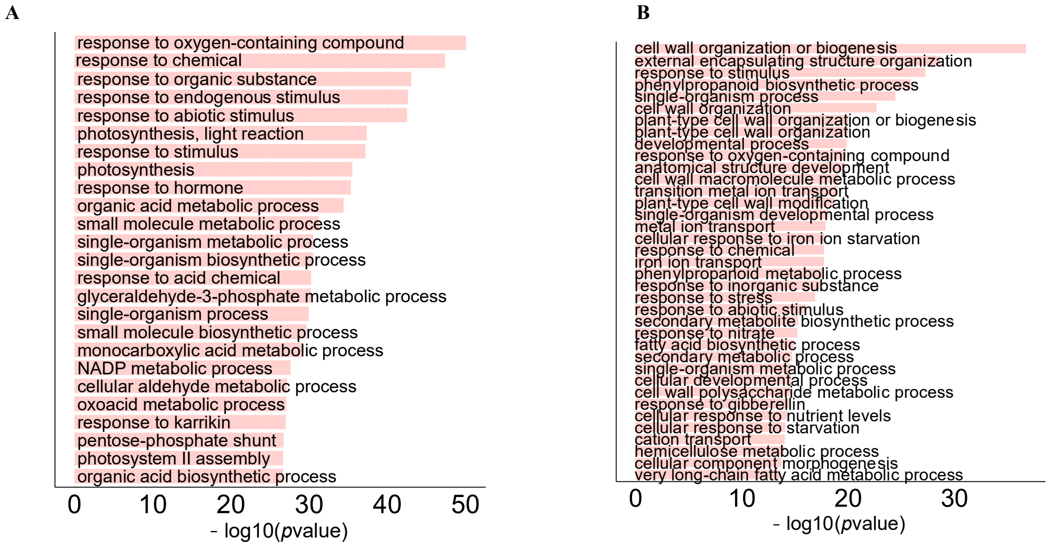

Figure S7. GO enrichment analysis of core genes marked by H3K4/K27me3 in C7(A) and C8 (B)

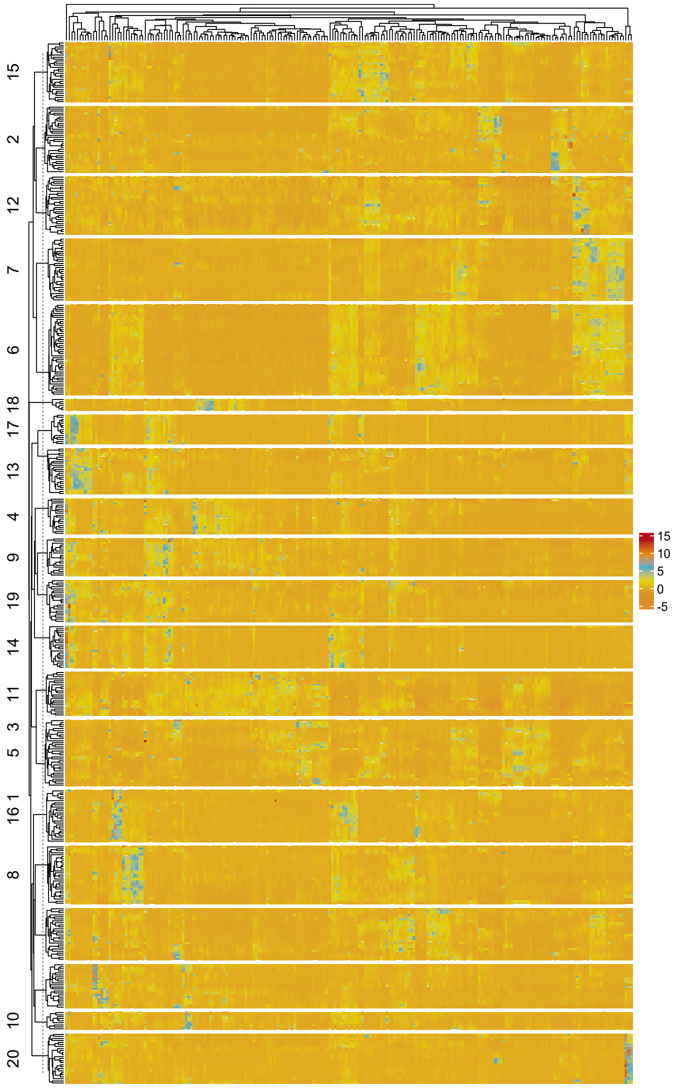

Figure S8. Heat map for clustering the expression values of the corresponding core TFs in C7 and C8 using the K-means method

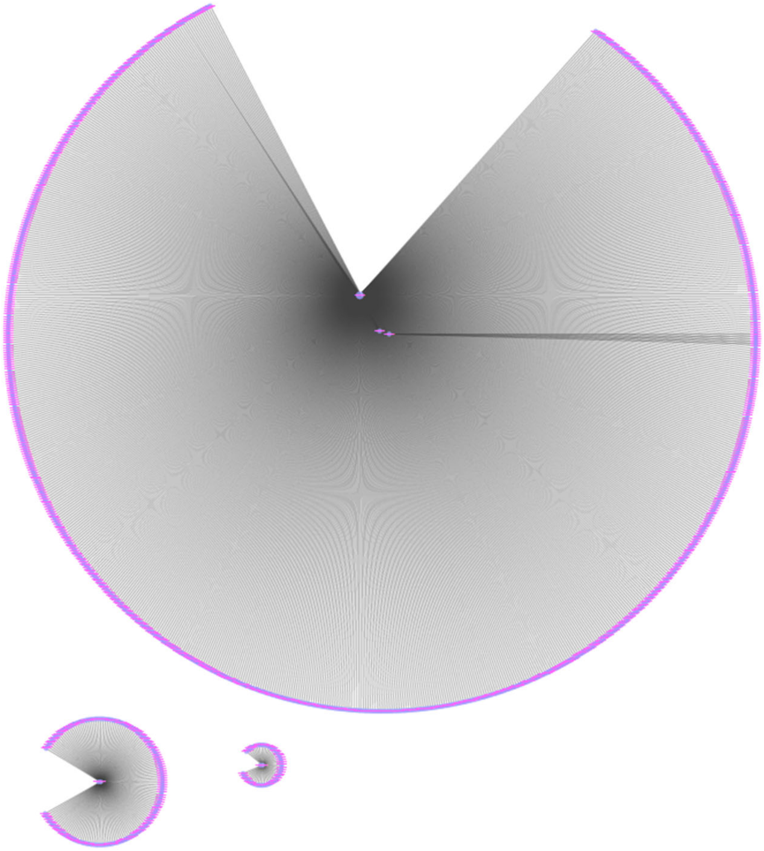

Figure S9. GRN network constructed by GENIE3 with top 1000 highest weighted regulatory relationships were selected and visualized by Cytoscape**.**
